# Supplementary material for: Shared Treg dysregulation underlies psoriasis vulgaris and atopic dermatitis through the PHF19-PRC2 epigenetic axis
Source: Front Immunol. 2026 Aug 20;17:1932788. doi: 10.3389/fimmu.2026.1932788 (PMC13538097; doi:10.3389/fimmu.2026.1932788)
Supplement: Supplementary file 3 [file DataSheet3.zip › Additional files/Additional file 2/07.ZC3H15.forest.pdf]

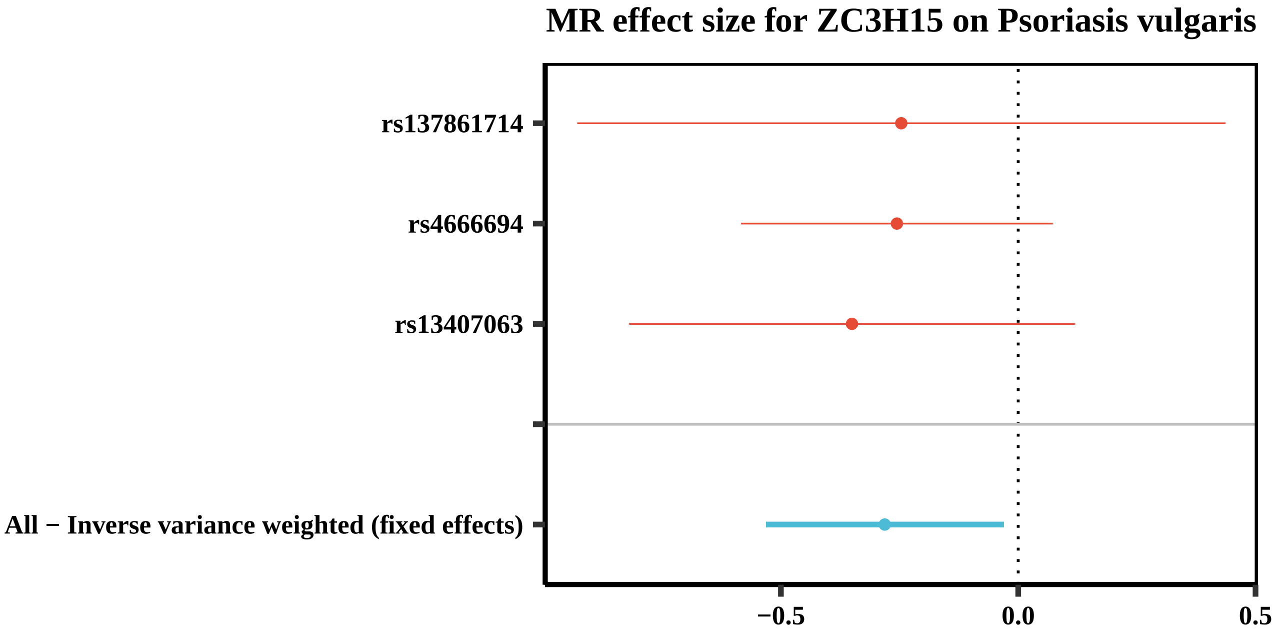
MR effect size for ZC3H15 on Psoriasis vulgaris
rs137861714
rs4666694
rs13407063
All − Inverse variance weighted (fixed effects)
−0.5
0.0
0.5
